# Molecular Docking and Molecular Dynamics Simulation Studies of Triterpenes from *Vernonia patula* with the Cannabinoid Type 1 Receptor

**DOI:** 10.3390/ijms22073595

**Published:** 2021-03-30

**Authors:** Md Afjalus Siraj, Md. Sajjadur Rahman, Ghee T. Tan, Veronique Seidel

**Affiliations:** 1 Department of Pharmaceutical Sciences, Daniel K. Inouye College of Pharmacy, University of Hawaii at Hilo, Hilo, HI 96720, USA; afjalus.siraj@gmail.com (M.A.S.); gheetan@hawaii.edu (G.T.T.); 2 Department of Chemistry and Biochemistry, South Dakota State University, Brookings, SD 57007, USA; sajjadur38@gmail.com; 3 Natural Products Research Laboratory, Strathclyde Institute of Pharmacy and Biomedical Sciences, University of Strathclyde, Glasgow G4 0RE, UK

**Keywords:** molecular docking, molecular dynamics, triterpenes, *Vernonia patula*

## Abstract

A molecular docking approach was employed to evaluate the binding affinity of six triterpenes, namely epifriedelanol, friedelin, α-amyrin, α-amyrin acetate, β-amyrin acetate, and bauerenyl acetate, towards the cannabinoid type 1 receptor (CB1). Molecular docking studies showed that friedelin, α-amyrin, and epifriedelanol had the strongest binding affinity towards CB1. Molecular dynamics simulation studies revealed that friedelin and α-amyrin engaged in stable non-bonding interactions by binding to a pocket close to the active site on the surface of the CB1 target protein. The studied triterpenes showed a good capacity to penetrate the blood–brain barrier. These results help to provide some evidence to justify, at least in part, the previously reported antinociceptive and sedative properties of *Vernonia patula*.

## 1. Introduction

The cannabinoid receptors (CB) belong to the superfamily of G protein-coupled receptors and are divided into two major types: CBR type-1 (CB1) and CBR type-2 (CB2). The CB1 receptors are commonly found in the central nervous system (CNS) and mostly control pain, movement, and neurotic parameters [1,2,3]. CB1 can be also found in peripheral tissues including retina [4], colon [5], testis [6], sperm cells [7], and adipocytes [8]. In contrast, CB2 receptors are mostly found in peripheral tissues [9]. Cannabinoids have already demonstrated great potential for the treatment of pain, inflammation, and neurodegenerative disorders [10,11,12]. These include the phytocannabinoids from *Cannabis sativa* and other plant-derived cannabinoid-like molecules called cannabimimetics that can interact with the endogenous cannabinoid system [13].

*Vernonia patula* (Dryand.) Merr. (Asteraceae) (VP) is an annual herb widely distributed throughout Southeast Asia. It is used medicinally for malaria, colds, fevers, respiratory ailments, and convulsions [14,15]. The leaves are used for their analgesic properties [16]. The aerial parts of VP have displayed antioxidant and anti-inflammatory activity [17]. Several *Vernonia* species, including VP, have also demonstrated antinociceptive and sedative properties [18,19,20]. The aerial parts of VP are known to contain flavonoids, simple phenolics, and terpenoids [21,22,23]. Previous reports have indicated that triterpenes could act as CB1 receptor agonists [24]. In the present work, we conducted molecular docking and molecular dynamics simulation studies on six triterpenes previously reported in the aerial parts of VP against the CB1 receptor with a view to (i) validating the medicinal properties of this plant and (ii) identifying new plant-derived cannabimimetic drug templates.

## 2. Results

### 2.1. HPLC-DAD-MS Analysis

The results of the HPLC-DAD-MS analysis (Figures S1–S3) indicated the presence of epifriedelanol (**1**), friedelin (**2**), α-amyrin (**3**) and α-amyrin acetate (**4**), β-amyrin acetate (**5**), and bauerenyl acetate (**6**) in VP.

### 2.2. Prediction of the Pharmacokinetic and Drug-Likeness Properties

All triterpenes had a molecular weight of less than 500 g/mol. Their Moriguchi LogP (MLogP) values were higher than those of the tetrahydrocannabinol (THC) control, while their polar surface areas were lower than THC (Table 1). All compounds showed high lipophilicity and insolubility in the Bioavailability Radar plots (Figure S4). Friedelin and α-amyrin demonstrated a similar human intestine absorption prediction score to that of THC (0.99). All triterpenes showed a good capacity to penetrate the blood–brain barrier. Friedelin showed the highest blood–brain barrier penetration prediction score (0.99), comparable to THC (1.00).

### 2.3. Molecular Docking

**Figure 1:**
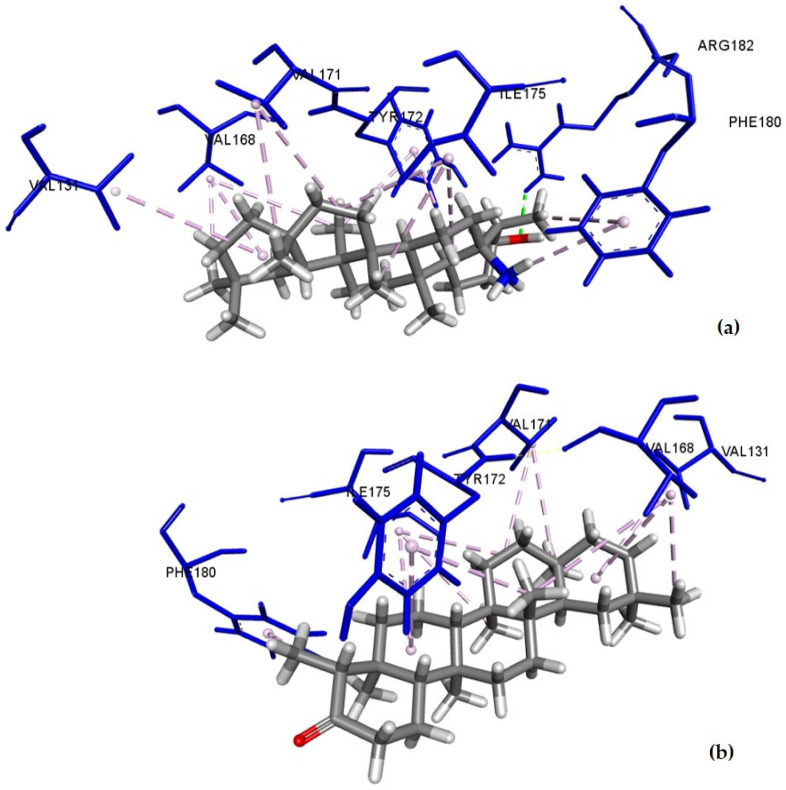

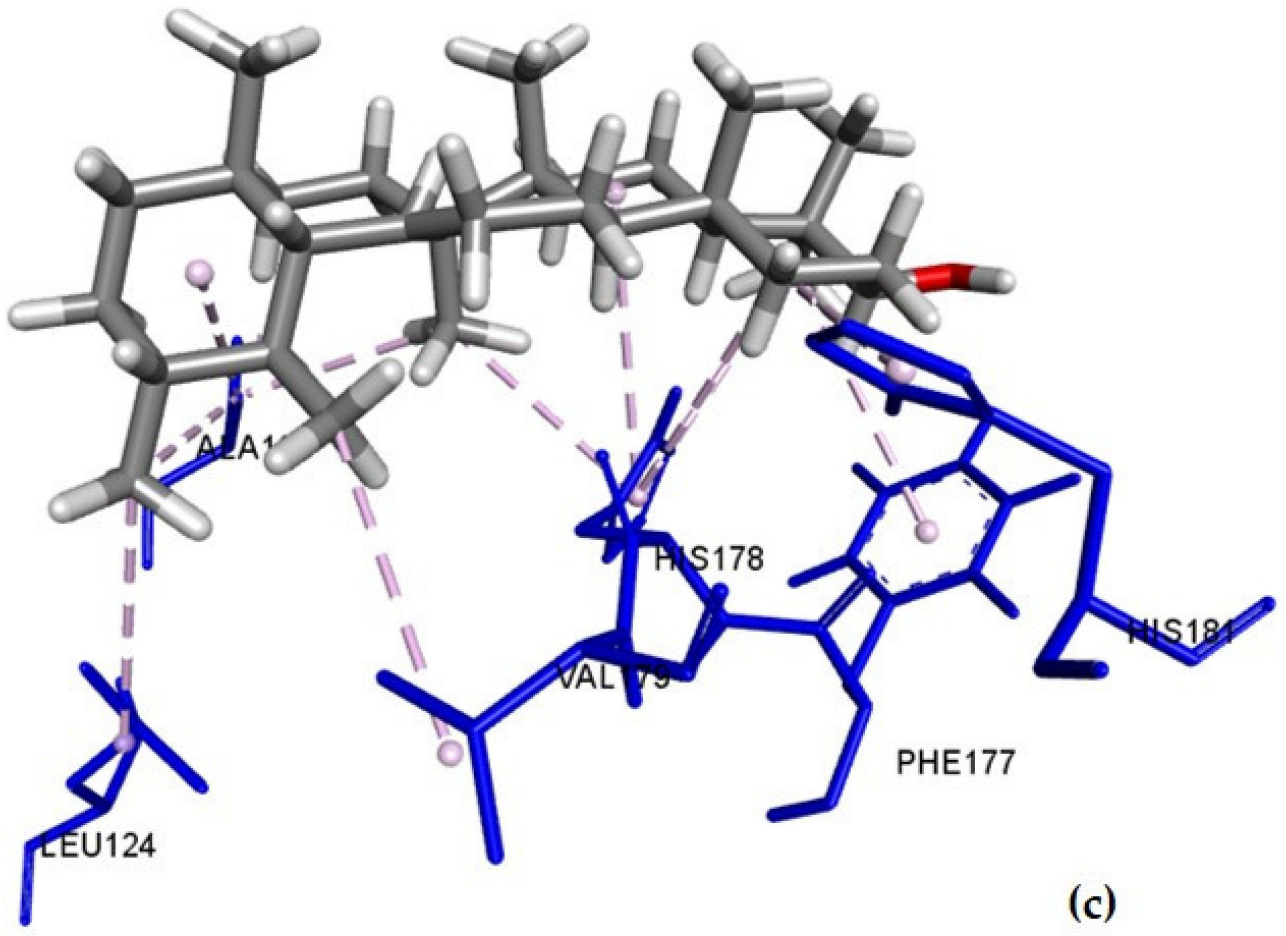
Docked pose of (**a**) epifriedelanol; (**b**) friedelin; (**c**) α-amyrin in the CB1 binding site showing molecular interactions—hydrogen and hydrophobic bonds as green and pink/purple dashed lines, respectively, generated by BIOVIA Discovery Studio visualizer.

Alpha-amyrin, friedelin, and epifriedelanol displayed the best binding affinities for CB1 with scores of −8.3, −8.1, and −8.1 kcal/mol, respectively. The THC control had a predictive binding affinity of −9.4 kcal/mol. The amino acid residues of CB1 involved in the binding with THC, epifriedelanol (**1**), friedelin (**2**), and α-amyrin (**3**) with their bonding distances are depicted in Table 2. The predictive binding affinity of the remaining triterpenes towards CB1 are shown in Table S1. The intermolecular binding interactions of epifriedelanol–CB1, friedelin–CB1, and α-amyrin–CB1 are depicted in Figure 1. THC showed 16 interactions, including one conventional H-bond (THC-OH·Met384-S), one Pi-Sigma, one amide-Pi stacking, and six alkyl and seven Pi-Alkyl bonds. It formed non-bonding interactions with both Phe102 and Phe379 residues within the active site of the CB1 protein. Epifriedelanol formed one conventional H-bond (Arg182-NH_2_·Epifriedelanol-O), and 13 hydrophobic (9 alkyl and 4 Pi-alkyl) non-bonding interactions. Friedelin formed 13 hydrophobic (9 alkyl and 4 Pi-alkyl) non-bonding interactions. Both epifriedelanol and friedelin were found to bind to a pocket containing residues Ile175, Tyr172, and Phe180 that were close to the active binding site of the THC control. Alpha-amyrin formed 12 hydrophobic (6 alkyl and 6 Pi-alkyl) non-bonding interactions with residues Ala120, Phe177, His178, and Val179, also close to the active binding site.

### 2.4. Molecular Dynamics (MD) Simulations

#### 2.4.1. Total Potential Energy Calculations

**Figure 2:**
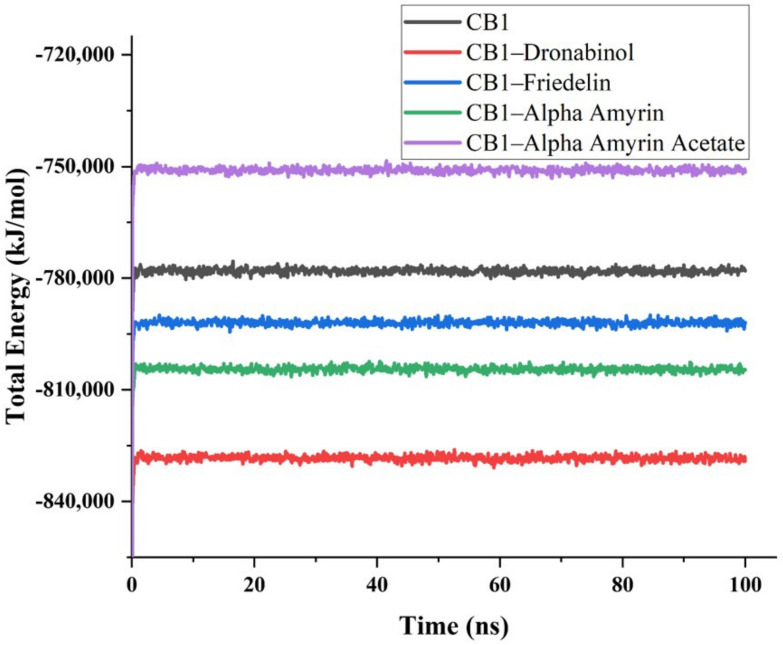
Total potential energies (kJ/mol) of the CB1 protein and the CB1–ligand complexes as a function of time.

Molecular dynamics simulations were conducted to further analyze the stability and binding affinities of the triterpene–CB1 complexes compared to the THC–CB1 complex. As both friedelin and epifriedelanol are structurally similar, only friedelin was selected for molecular dynamics simulations. Alpha-amyrin was also considered as it showed the best docking score among all triterpenes. Alpha-amyrin acetate was selected as a representative for acetate-containing compounds. The total potential energies of CB1, CB1–THC, CB1–friedelin, CB1–α-amyrin, and CB1–α-amyrin acetate were calculated for a period of 100 ns. Values obtained for the energy-minimized initial conformations at 0 ps were −1024595.105, −1090152.423, −1045113.241, −1056698.855, and −992631.612 kJ/mol, respectively. The values obtained for each studied system after a few picoseconds were −790249.746, −842086.509, −804131.370, −815489.160, and −761594.499 kJ/mol, respectively. The total potential energies of the studied systems remained stable throughout the 100 ns simulation period (Figure 2). The CB1–THC complex showed the lowest potential energy, successfully binding at the active site of CB1. The triterpenes were found to bind strongly to a pocket at the surface of the target protein, close to the active binding site of THC. The CB1–friedelin and CB1–α-amyrin complexes showed lower potential energy values than CB1 alone. The CB1–α-amyrin acetate complex showed higher energy than CB1 alone. All complexes were stable and in compliance with the docking scores obtained.

#### 2.4.2. Principal Components Analysis of MD Simulations

**Figure 3:**
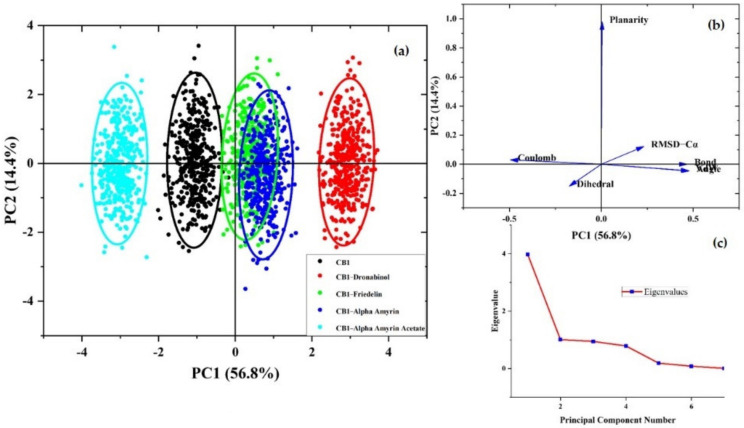
(**a**) Score plot presenting five data clusters in a different color, where each dot represents the one-time point. The clustering is attributable as CB1 (black), THC–CB1 (red), friedelin–CB1 (green), α-amyrin–CB1 (blue), and α-amyrin acetate–CB1 (turquoise); (**b**) loading plot generated from the MD energy profiles and structural data; (**c**) scree plot with eigenvalues.

The PCA scores plot reveals different clusters formation of the protein and the protein–ligand complexes based on their structure and energy profiles (Figure 3a). The cluster for the α-amyrin–CB1 complex overlapped the one for the friedelin–CB1 complex. On the PC2 axis, the friedelin–CB1 cluster showed a similar pattern to the CB1 cluster, but more compactness. The α-amyrin acetate–CB1 complex formed a distinct cluster showing negative correlation on the PC1 axis differing from the other triterpene–CB1 and THC–CB1 complexes. The cluster of the THC–CB1 complex was further away from the CB1 cluster compared to the other complexes. The loading plot generated from the molecular dynamics (MD) energy profiles, and structural data showed the bond, angle, and Van der Waals energies and RMSD-Cα values as the major contributing factors for the stabilizing of the protein and the complexes (Figure 3b). A scree plot showing the eigenvalues further validated the analyses (Figure 3c).

#### 2.4.3. Stability Analysis

**Figure 4:**
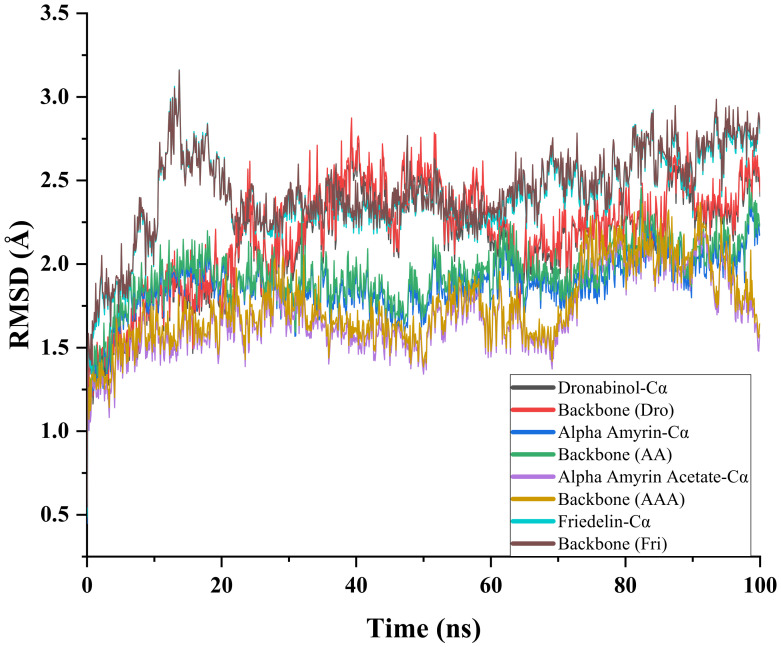
Time series of the RMSD of alpha-carbon atoms (Cα) and of the whole backbone atoms for CB1 and CB1–ligand complexes.

The atomic root mean square deviations (RMSDs) of Cα atoms of CB1 and of the selected CB1–triterpene complexes were analyzed to compare their structural stability (Figure 4). All complexes reached equilibrium after 50 ns. The α-amyrin acetate–CB1 complex exhibited the lowest RMSD value among all complexes. A decrease in RMSD was observed for this complex from 80 to 100 ns, suggesting that this complex may not be stable overall. The RMSD value of the α-amyrin–CB1 complex showed a more stable pattern than the corresponding acetate complex. The RMSD values obtained for the α-amyrin–CB1 and the α-amyrin acetate–CB1 complexes were lower than those of CB1 alone, which suggest that α-amyrin and α-amyrin acetate contributed to lower the energy of the protein and made the protein more stable. The RMSDs of the CB1–THC and CB1–friedelin complexes fluctuated greatly from 10 to 60 ns and then stabilized. The value of the CB1–THC complex reached 2.0–2.2 Å after 60 ns. The RMSD of the CB1–friedelin complex fluctuated mostly between 10 and 20 ns, then showed a steady increase over the simulation period. These relatively higher deviations compared to the RMSD of the CB1 structure suggested that THC and friedelin may change the protein conformation at the binding site.

#### 2.4.4. Residue Mobility Analysis

**Figure 5:**
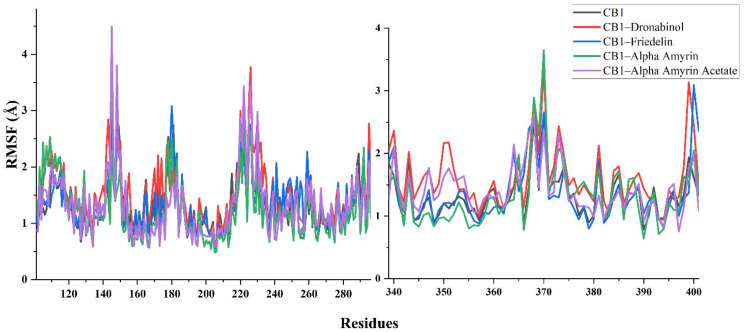
Structural behavioral changes of the CB1 protein by RMSF per residue with a focus on 101–300 and 333–401 residues.

The binding of THC to CB1 was found to primarily induce local flexibility of the active site residues and revealed that the protein became more flexible in all regions. In contrast, the α-amyrin–CB1 and the friedelin–CB1 complexes showed the lowest RMSF, indicating that the binding of α-amyrin and friedelin to CB1 made the protein less flexible. The α-amyrin acetate–CB1 complex showed a similar trend to the THC–CB1 complex in terms of residue motility analysis (Figure 5).

### 2.5. MM-PBSA Binding Free Energy Analysis

**Figure 6:**
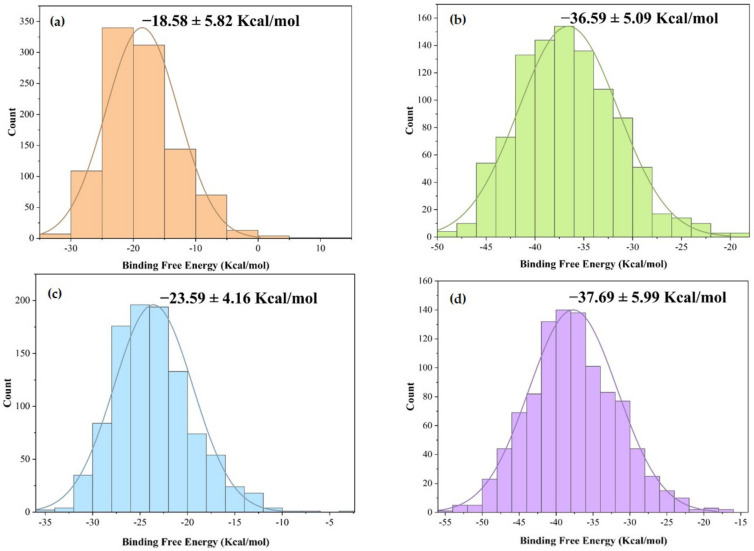
Histogram of the binding free energy value obtained for (**a**) CB1–α-amyrin; (**b**) CB1–α amyrin acetate; (**c**) CB1–friedelin; (**d**) CB1–THC, where bell-shaped curves represent a Gaussian fit.

Additional calculations of the binding free energies of the CB1–ligand complexes investigated in the molecular dynamics simulations using the molecular mechanics Poisson–Boltzmann surface area (MM-PBSA) method are presented in Figure 6. Alpha-amyrin acetate showed the highest binding free energy value (−36.6 ± 5.09 Kcal/mol) among the selected triterpenes.

## 3. Discussion

It has previously been demonstrated that the aerial parts of VP are antinociceptive and can induce sedation by suppressing locomotor activity and exploratory behavior in mice. This has been linked with the presence of phenolic compounds predicted to interact with CB1 [20,23,25]. However, it has also been reported that triterpenes, including α-amyrin and β amyrin, could induce in vivo antinociceptive and anti-inflammatory effects via activation of the cannabinoid receptors [24,26]. Other terpenoids with effects on the CNS include α-pinene, which suppresses locomotor activity, increases sleeping time, and produces an anxiolytic effect in vivo [27], and phytol and terpinolene with sedative and locomotor suppressive effects [28,29]. Similar sleep-inducing and locomotor relaxant effects have been reported for citral, limonene, and myrcene [30]. A decrease in locomotor activity can be correlated to a potential CNS depression [31]. Myrcene and α-pinene have been shown to increase the GABA_A_ receptor activity in vitro and potentiate the release of inhibitory neurotransmitters [32]. Moreover, it was also reported that GABA_A_-stimulating drugs such as flurazepam can synergistically potentiate the cataleptic effects of THC in mice [33].

The purpose of our computational studies was to evaluate the predictive binding affinity of VP triterpenes towards the CB1 receptor and carry out molecular dynamics simulations to describe the nature of the interactions of these triterpenes with CB1. Friedelin, α-amyrin, and epifriedelanol showed a strong binding affinity for the CB1 receptor in our molecular docking study. Molecular dynamics simulations, through stability and residue mobility analyses, was used to understand the structural variations and conformational flexibility of the CB1 protein and CB1 complexed with the selected triterpenes. All the studied triterpenes showed stable binding throughout the molecular dynamics simulations. The most stable interactions with CB1 were predicted for α-amyrin and friedelin. The latter had a blood–brain barrier penetration prediction score comparable to THC. Our molecular docking and molecular dynamics simulation results suggest that by interacting with CB1 receptors, VP triterpenes could contribute to the significant antinociceptive and CNS-depressant activity we have previously demonstrated for this plant [20].

## 4. Materials and Methods

### 4.1. Plant Collection and Extraction

The aerial parts of VP were collected from Chittagong (Bangladesh) in 2015. The plant material was identified by M.A. Ali at the Bangladesh National Herbarium in Dhaka where a voucher specimen (DACB: 35107) is kept for future reference. The dried powdered aerial parts of VP (100 g) were macerated in ethyl acetate (EtOAc; 1.5 L) for 5 days at 25 ± 2 °C, with occasional shaking. The resulting filtrate was concentrated under reduced pressure to afford the final extract (6.2 g, 6.2% yield).

### 4.2. HPLC-DAD-MS Analysis

The extract (10 mg) was subjected to solid phase extraction (SPE) using a Hypersep C18 cartridge (Thermo Scientific, Waltham, MA, USA) eluting sequentially with 70, 80, 90, and 100% methanol (MeOH). Fraction 1 (1.5 mg), fraction 2 (1.8 mg), fraction 3 (2.1 mg), and fraction 4 (4.2 mg) were collected, and stock solutions (0.1 mg/mL) were prepared for further analysis. The sample solution and blank control were all stored at 4 °C and filtered through a 0.22 μm PTFE syringe filter (Thermo Scientific, Waltham, MA, USA) prior to analysis by HPLC-DAD-MS. Analysis was carried out using an Agilent HPLC 1260 binary pump and a Sunfire C18 column (2.1 × 150 mm, 3.5 µm) (Waters, Milford, MA, USA). The diode array detector (DAD) was set at λ = 210, 254, 269, and 310 nm. The known, or suspected, major adducts for all samples were registered in the positive electrospray ionization (ESI) mode either as [M+H]^+^ or [M+Na]^+^. The mobile phase used was a gradient of 1% formic acid in water and acetonitrile (solvents A and B, respectively) at a flow rate of 0.2 mL/min. Elution was performed as follows; Fraction 1 (0 to 30 min—60 to 80% solvent B), fraction 2 (0 to 30 min—70 to 90% solvent B), fraction 3 (0 to 30 min—80 to 100% solvent B), and fraction 4 (0 to 30 min—100% solvent B).

### 4.3. Prediction of Pharmacokinetic and Drug-Likeness Properties

Absorption, distribution, metabolism, excretion, and toxicity (ADMET) properties of the selected triterpenes were determined using the online ADMET structure-activity relationship database (admetSAR). The specific pharmacokinetic profile for each compound was obtained using compound specific SMILES strings [34]. MLogP was used as an alternative to the regular LogP model [35]. In addition, drug-likeness and molecular properties of the triterpenes were calculated using SwissADME, and Bioavailability Radar plots were generated taking account of lipophilicity, size, polarity, solubility, flexibility, and saturation [36].

### 4.4. Molecular Docking

#### 4.4.1. Ligands Optimization

As triterpenes had been reported as CB1 receptor agonists [23], we decided to consider six triterpenes, namely epifriedelanol (**1**), friedelin (**2**), α-amyrin (**3**), α-amyrin acetate (**4**), β-amyrin acetate (**5**), and bauerenyl acetate (**6**) (Figure S5), previously reported in the aerial parts of VP [4,5] as the putative ligands for our in silico analyses. Optimizations for the triterpenes and vibrational frequency calculations were determined at gaseous phase using the Gaussian 09 software version A.02 [37] with a semi-empirical PM6 method [38]. The three dimensional structures of each compound were obtained from the PubChem database (https://pubchem.ncbi.nlm.nih.gov/, accessed on 26 March 2021), geometry-optimized, and saved in PDB format using GaussView v.5.0 (https://gaussian.com/gaussview6/, accessed on 26 March 2021).

#### 4.4.2. Protein Optimization

The crystal structure of the human CB1 cannabinoid receptor and its active site [39,40] was retrieved from the RSCB Protein Data Bank (PDB ID: 5U09). The heteroatoms and water molecules were removed from the crystal structure using PyMOL Molecular Graphics System v. 1.3 (https://pymol.org, accessed on 26 March 2021) [41]. The structure of the protein was further optimized using the Swiss-PDB viewer software (v.4.1.0) based on the energy minima. The protein and ligand structures were saved in the PDBQT format.

#### 4.4.3. Determination of Ligand-Protein Binding Affinities and Non-Bonding Interactions

The active binding pocket of CB1 was predicted by CASTp (v. 3.0) [42]. The protein showed the highest pocket area and volume at 652.65 Å^2^ and 331.44 Å^3^, respectively. The grid box was generated so as to include the CB1 binding site residues, with a center set at 12.5230, 7.2495, and 17.7743 Å and a size set at 81.5, 81.5, and 81.5 Å in *x*, *y*, and *z* directions, respectively. Autodock Vina (v.1.1.2) was used to perform the molecular docking study [43]. The docked pose of the lowest binding free energy conformer (highest probable binding affinity) with CB1 was analyzed using PyMOL Molecular Graphics System (v. 1.3) (https://pymol.org, accessed on 26 March 2021), Drug Discovery Studio (v. 4.1) (https://www.3dsbiovia.com, accessed on 26 March 2021), and LigPlot+ (v. 1.4.5) (https://www.ebi.ac.uk/thornton-srv/software/LigPlus/, accessed on 26 March 21).

### 4.5. Molecular Dynamics Simulations

#### 4.5.1. Total Potential Energy Calculations

The molecular dynamics (MD) simulations were performed on the CB1, CB1–THC, and three selected triterpene–CB1 complexes, namely CB1–friedelin, CB1–α-amyrin, and CB1–α-amyrin acetate. The first two complexes were chosen based on the highest docking scores obtained in the molecular docking study. The third complex was selected to observe the influence of an acetate group on the protein–ligand interactions. MD simulations were conducted using YASARA Dynamics v. 20.8.1 [44]. The AMBER14 force field was employed to simulate the macromolecular system [45]. Each protein was subject to hydrogen bond optimization prior to simulation [44], and the transferable intermolecular potential 3-point (TIP3P) water model was used by incorporating Cl− and/or Na+ ions. Periodic boundary conditions were incorporated to perform the simulations, where the cell size was 10 Å larger than the protein size in all cases. The initial energy minimization for each system was performed using the steepest gradient approach (5000 cycles), MD simulations were carried out using the particle-mesh Ewald (PME) method to designate long-range electrostatic interactions at a cut off distance of 8 Å and defining physiological conditions at 298 °K, pH 7.4, 0.9% NaCl [46]. The simulation temperature was controlled using a Berendsen thermostat with the pressure kept constant. A multiple time step algorithm was employed with a time step of 2.00 fs [47]. Finally, MD simulations were performed for 100 ns at constant pressure and Berendsen thermostat, and snapshots were saved every 100 ps. Further analysis was conducted using the default YASARA MACRO script [48].

#### 4.5.2. Principal Component Analysis of MD Simulation Data

Principal component analysis (PCA) was used to analyze any subtle variability among the structural and energy profile data obtained from MD simulations for the selected triterpene–CB1 complexes and CB1 alone. Bond energies, bond angle energies, dihedral angle energies, planarity energies, Coulomb energies, Van der Waals energies, and RMSD-Cα values were included as the variables [49,50,51]. Multivariate responses were arranged in an *X* matrix according to the following equation:

(1) $$X=T_{k}P_{k}^{T}+E$$

where *T_k_* describes how the samples relate to each other, *P_k_* demonstrates how the variables relate to each other, *k* is the number of factors included in the model, and *E* is the matrix of residuals. PCA was conducted using the OriginPro 2021 (Principal Component Analysis app v.1.50) software package.

#### 4.5.3. Stability and Residue Mobility Analyses

The root mean square deviations (RMSDs) of Cα atoms on the backbone of the CB1 protein and of the selected CB1–triterpene complexes were calculated during the MD simulation period using YASARA macro file followed by OriginPro 2021. Root mean square fluctuation (RMSF) analysis using YASARA macro script and OriginPro 2021 was used to observe the regions that fluctuated during the MD simulation period.

### 4.6. MM-PBSA Binding Free Energy Calculations

The molecular mechanics Poisson–Boltzmann surface area (MM-PBSA) method [52] was used to calculate the binding free energies of the CB1–ligand complexes investigated in the molecular dynamics simulations. Default macro scripts of YASARA dynamics were employed for the calculations. Selected snapshots from the last 50 ns MD simulation were used for all CB1–ligand complexes. Protein ligand binding free energy values were calculated using the following equation:

ΔG_binding_ = ΔG_complex_ − [ΔG_ligand_ + ΔG_protein_], and
ΔG_binding_ = ΔG_MM_ + ΔG_PB_+ΔG_SA–TΔS_ = (ΔG_elec_+ ΔG_VdW_) + ΔG_PB_ + ΔG_SA–TΔS_ (2)

where ΔG_complex_ = total free energy of the protein–ligand complex in solvent, ΔG_ligand_ = total energy of the ligand in solvent, and ΔG_protein_ = total energy of the protein in solvent. ΔG_MM_ = molecular mechanics interaction energy, where the ΔG_elec_ and ΔG_VdW_ are the electrostatic and Van der Waals interactions, respectively. ΔG_PB_ and ΔG_SA_ represent polar solvation and nonpolar solvation energy, respectively. TΔS (temperature = T and entropy = S) is the contribution of entropy to the free energy.

## Figures and Tables

**Table 1:** Drug-likeness parameters prediction for tetrahydrocannabinol (THC), epifriedelanol (**1**), friedelin (**2**), α-amyrin (**3**) and α-amyrin acetate (**4**), β-amyrin acetate (**5**), and bauerenyl acetate (**6**).

Parameters	THC	(1)	(2)	(3)	(4)	(5)	(6)
Structural formula	C_21_H_30_O_2_	C_30_H_52_O	C_30_H_50_O	C_30_H_50_O	C_32_H_52_O_2_	C_32_H_52_O_2_	C_32_H_52_O_3_
Molecular weight (g/mol)	314.46	428.73	426.72	426.72	468.75	468.75	468.75
Blood–brain barrier (p.s.) ^a^	1.00	0.96	0.99	0.84	0.83	0.83	0.83
MLogP ^b^	4.39	7.07	6.92	6.92	7.08	7.08	7.08
TPSA ^c^ (Å^2^)	29.46	20.23	17.07	20.23	26.30	26.30	26.30
Human intestinal absorption (p.s.)	0.99	0.99	0.99	0.99	0.99	0.99	0.99
Human oral bioavailability	NSP ^d^	0.5857	0.6857	NSP	NSP	NSP	NSP
Bioavailability score	0.55	0.55	0.55	0.55	0.55	0.55	0.55

^a^ p.s. = prediction score. ^b^ MLogP = Log P_oil/water_. ^c^ TPSA = topological polar surface area. ^d^ NSP = negative score prediction.

**Table 2:** Intermolecular interactions of THC, epifriedelanol, friedelin, and α-amyrin with CB1.

Ligand	Binding Affinity (kcal/mol)	Binding Residues	Category	Type	Distance (Å)
THC	−9.4	Met384	Hydrogen Bond	Conventional	2.59
		Met103	Hydrophobic	Pi-Sigma	3.67
		Met103	Hydrophobic	Amide-Pi Stacked	4.71
		Ile105	Hydrophobic	Alkyl	4.27
		Leu111	Hydrophobic	Alkyl	5.12
		Ile119	Hydrophobic	Alkyl	4.54
		Val196	Hydrophobic	Alkyl	3.97
		Ala380	Hydrophobic	Alkyl	4.16
		Met103	Hydrophobic	Alkyl	4.93
		Ile105	Hydrophobic	Pi-Alkyl	5.19
		Phe102	Hydrophobic	Pi-Alkyl	5.24
		Phe102	Hydrophobic	Pi-Alkyl	4.70
		Phe108	Hydrophobic	Pi-Alkyl	4.16
		Phe108	Hydrophobic	Pi-Alkyl	3.76
		His178	Hydrophobic	Pi-Alkyl	4.76
		Phe379	Hydrophobic	Pi-Alkyl	5.15
Friedelin	−8.1	Val171	Hydrophobic	Alkyl	4.65
		Ile175	Hydrophobic	Alkyl	4.45
		Val171	Hydrophobic	Alkyl	4.81
		Val168	Hydrophobic	Alkyl	4.01
		Ile175	Hydrophobic	Alkyl	5.14
		Val168	Hydrophobic	Alkyl	5.34
		Val131	Hydrophobic	Alkyl	5.26
		Val168	Hydrophobic	Alkyl	4.3
		Ile175	Hydrophobic	Alkyl	5.43
		Tyr172	Hydrophobic	Pi-Alkyl	4.82
		Tyr172	Hydrophobic	Pi-Alkyl	4.64
		Phe180	Hydrophobic	Pi-Alkyl	4.93
		Phe180	Hydrophobic	Pi-Alkyl	4.69
α-amyrin	−8.3	Ala120	Hydrophobic	Alkyl	4.27
		Leu124	Hydrophobic	Alkyl	4.83
		Val179	Hydrophobic	Alkyl	4.74
		Ala120	Hydrophobic	Alkyl	4.03
		Ala120	Hydrophobic	Alkyl	4.20
		Ala120	Hydrophobic	Alkyl	3.88
		Phe177	Hydrophobic	Pi-Alkyl	4.98
		His178	Hydrophobic	Pi-Alkyl	4.95
		His178	Hydrophobic	Pi-Alkyl	4.96
		His178	Hydrophobic	Pi-Alkyl	4.80
		His178	Hydrophobic	Pi-Alkyl	4.22
		His181	Hydrophobic	Pi-Alkyl	4.41
Epifriedelanol	−8.1	Arg182	Hydrogen Bond	Conventional	2.32
		Val171	Hydrophobic	Alkyl	4.73
		Ile175	Hydrophobic	Alkyl	4.53
		Val171	Hydrophobic	Alkyl	4.93
		Val168	Hydrophobic	Alkyl	4.11
		Ile175	Hydrophobic	Alkyl	5.20
		Val168	Hydrophobic	Alkyl	5.37
		Val131	Hydrophobic	Alkyl	5.37
		Val168	Hydrophobic	Alkyl	4.30
		Ile175	Hydrophobic	Alkyl	5.46
		Tyr172	Hydrophobic	Pi-Alkyl	4.76
		Tyr172	Hydrophobic	Pi-Alkyl	4.62
		Phe180	Hydrophobic	Pi-Alkyl	4.76
		Phe180	Hydrophobic	Pi-Alkyl	4.88

## Data Availability

All data generated or analyzed during this study are included in this published article (and its Supplementary Information files).

## Supplementary Materials

The following are available online at https://www.mdpi.com/1422-0067/22/7/3595/s1.
