# Supplementary material for: Molecular Docking and Molecular Dynamics Simulation Studies of Triterpenes from Vernonia patula with the Cannabinoid Type 1 Receptor
Source: Int J Mol Sci. 2021 Mar 30;22(7):3595. doi: 10.3390/ijms22073595 (PMC8038099; doi:10.3390/ijms22073595)
Supplement: Supplementary file 1 [file ijms-22-03595-s001.pdf]

International Journal of *Molecular Sciences*

MDPI

Supplementary Material

Molecular Docking and Molecular Dynamics Simulation Studies of Triterpenes from *Vernonia patula* with the Cannabinoid Type 1 Receptor

Md Afjalus Siraj [1], Md. Sajjadur Rahman [2], Ghee T. Tan [1] and Veronique Seidel [3,*]

[1] Department of Pharmaceutical Sciences, Daniel K. Inouye College of Pharmacy, University of Hawaii at Hilo, Hilo, HI 96720, USA; afjalus.siraj@gmail.com; masiraj@hawaii.edu; gheetan@hawaii.edu
[2] Department of Chemistry and Biochemistry, South Dakota State University, Brookings, SD 57007, USA; sajjadur38@gmail.com
[3] Natural Products Research Laboratory, Strathclyde Institute of Pharmacy and Biomedical Sciences, University of Strathclyde, Glasgow, UK; veronique.seidel@strath.ac.uk
* Correspondence: veronique.seidel@strath.ac.uk

Citation: Siraj, M.A; Rahman, M.S; Tan, G.T; Seidel, V. Molecular docking and molecular dynamics simulation studies of triterpenes from *Vernonia patula* with the cannabinoid type 1 receptor. *Int. J. Mol. Sci.* **2021**, *22*, 3595. https://doi.org/10.3390/ijms22073595

Academic Editor: Firstname Lastname
Received: 18 February 2021
Accepted: 23 March 2021
Published: 30 March 2021

Publisher's Note: MDPI stays neutral with regard to jurisdictional claims in published maps and institutional affiliations.

Copyright: © 2021 by the authors. Licensee MDPI, Basel, Switzerland. This article is an open access article distributed under the terms and conditions of the Creative Commons Attribution (CC BY) license (http://creativecommons.org/licenses/by/4.0/).

Abstract: A molecular docking approach was employed to evaluate the binding affinity of six triterpenes, namely epifriedelanol, friedelin, α-amyrin, α-amyrin acetate, β-amyrin acetate and bauerenyl acetate, towards the cannabinoid type 1 receptor (CB1). Molecular docking studies showed that friedelin, α-amyrin and epifriedelanol had the strongest binding affinity towards CB1. Molecular dynamics simulation studies revealed that friedelin and α-amyrin engaged in stable non-bonding interactions by binding to a pocket close to the active site on the surface of the CB1 target protein. The studied triterpenes showed a good capacity to penetrate the blood-brain barrier. These results help to provide some evidence to justify, at least in part, the previously reported antinociceptive and sedative properties of *Vernonia patula*.

Keywords: molecular docking; molecular dynamics; triterpenes; *Vernonia patula*

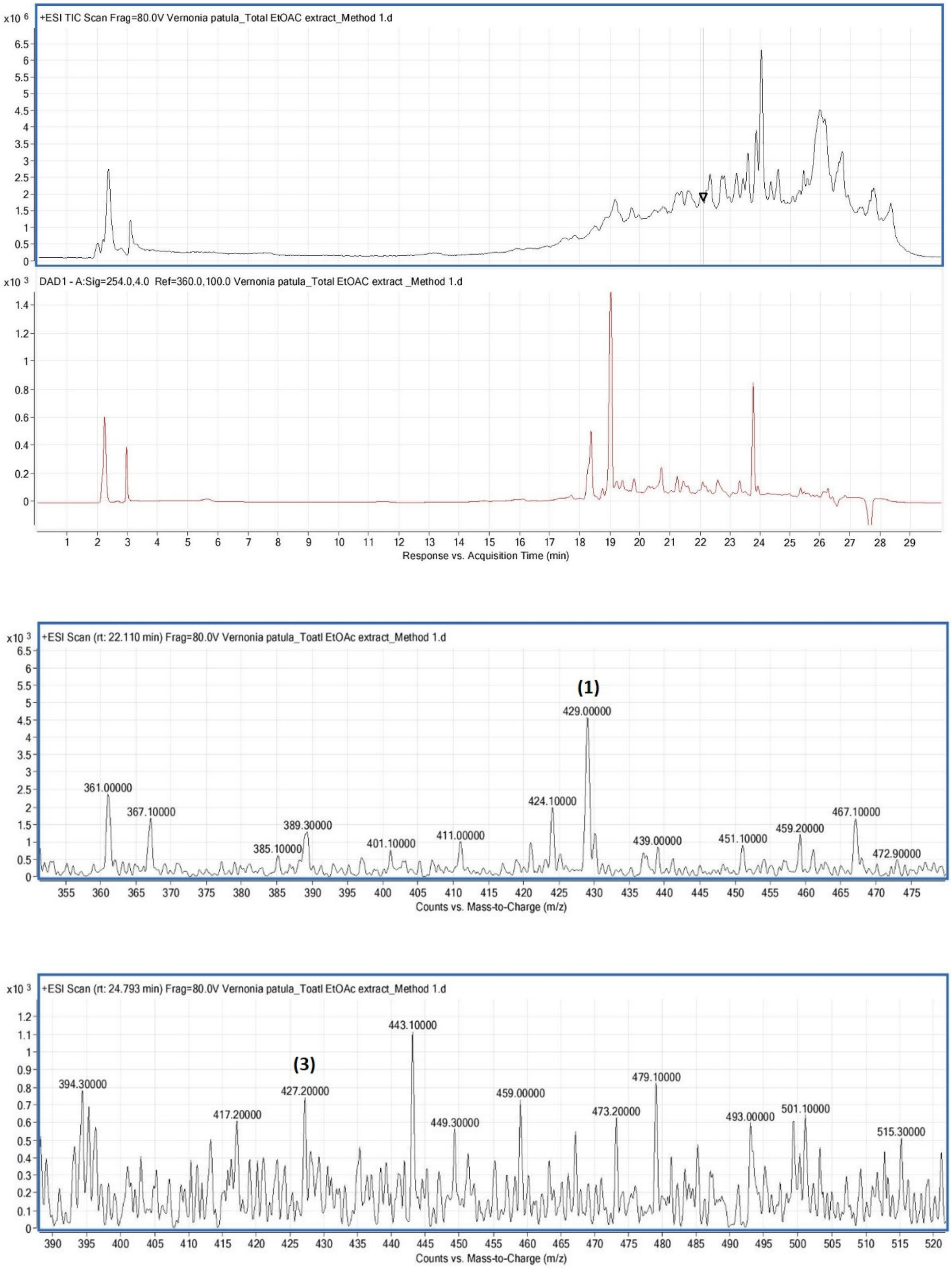

Figure 1. Detection of epifriedelanol (**1**) and α-amyrin (**3**) in VP (fraction 1) using HPLC-DAD-MS analysis.

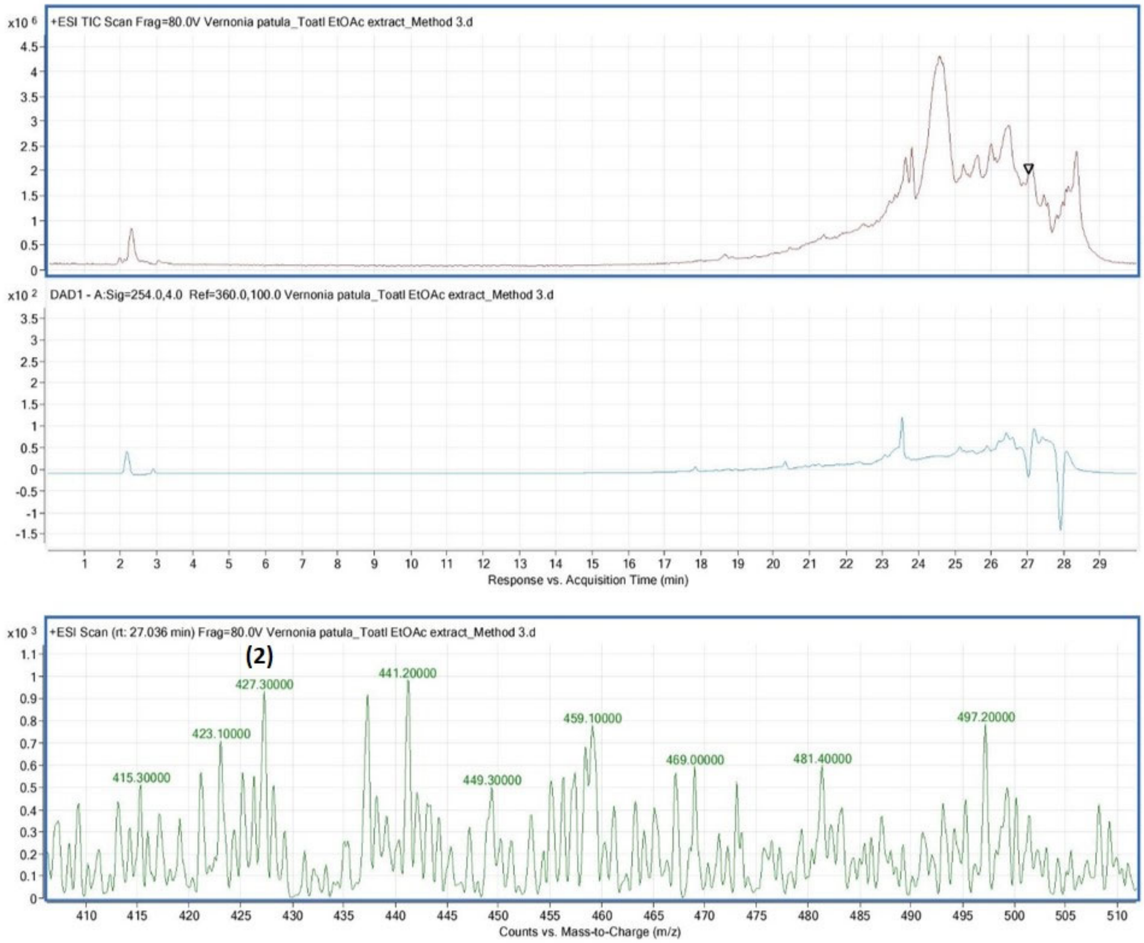

Figure 2. Detection of friedelin (**2**) in VP (fraction 3) using HPLC-DAD-MS analysis.

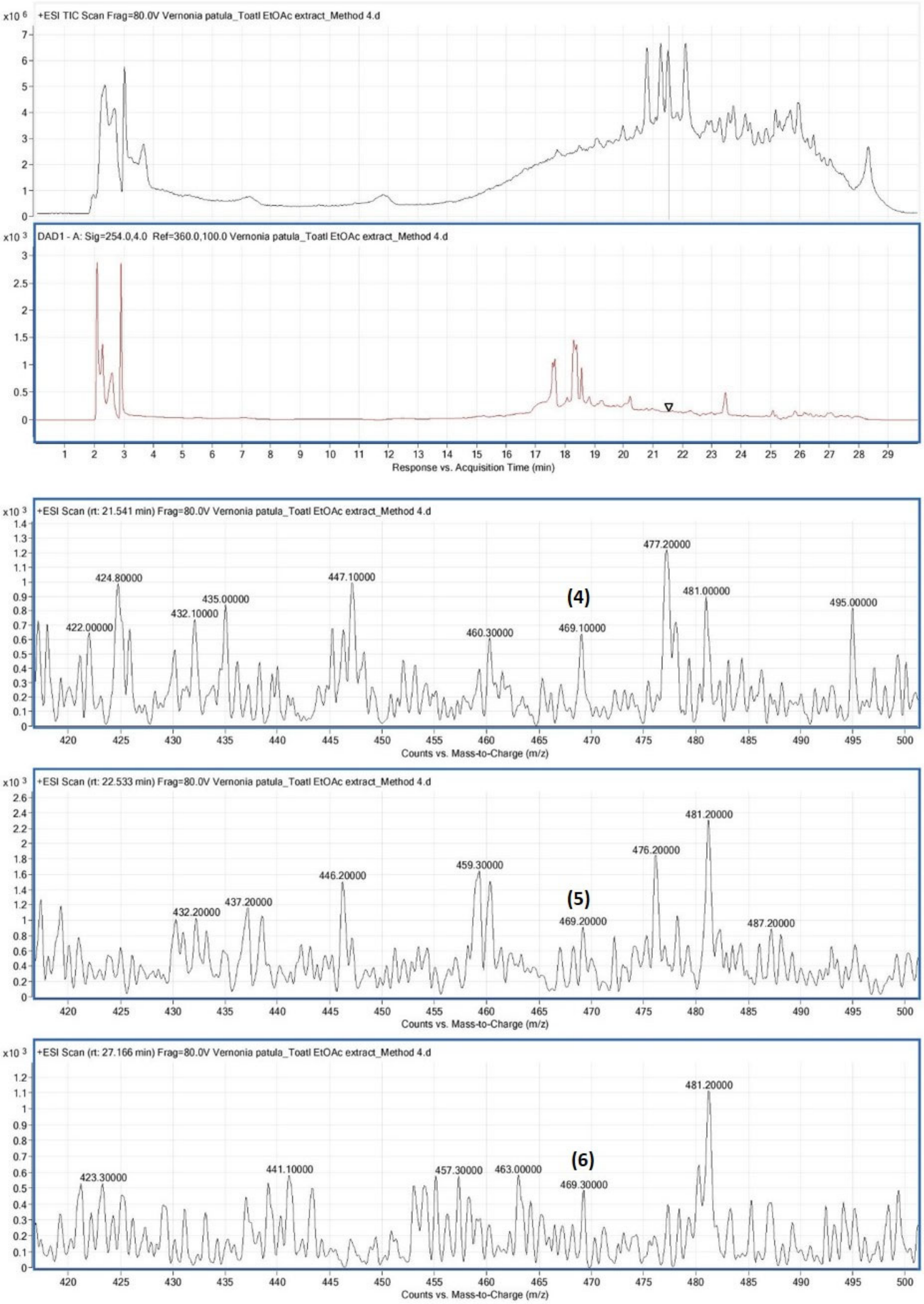

Figure 3. Detection of α-amyrin acetate (**4**), β-amyrin acetate (**5**) and bauerenyl acetate (**6**) in VP (fraction 4) using HPLC-DAD-MS analysis.

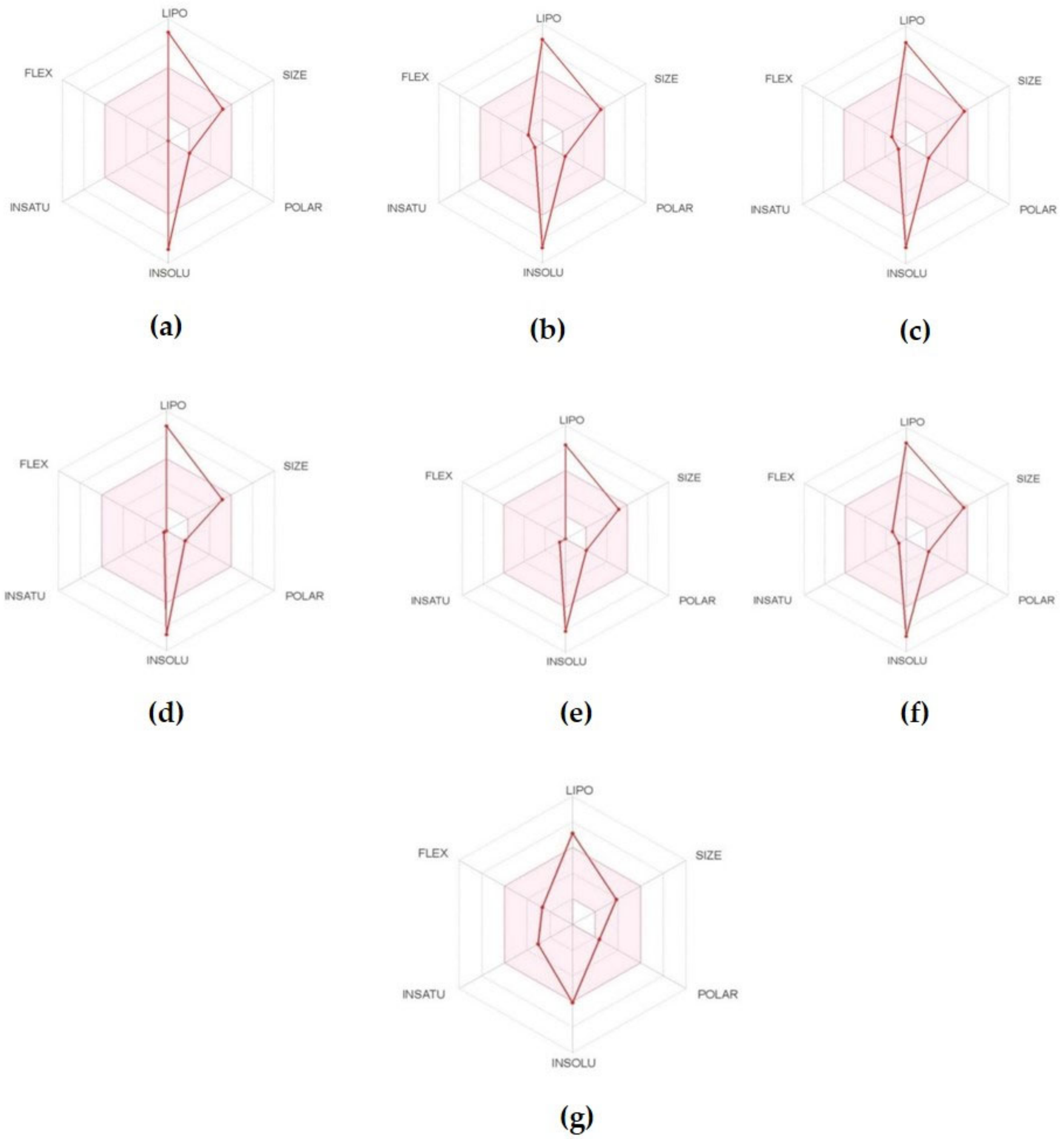

Figure 4. Bioavailability Radar plots of (**a**) epifriedelanol; (**b**) β amyrin acetate; (**c**) bauerenyl acetate; (**d**) friedelin; (**e**) α-amyrin; (**f**) α- amyrin acetate; (**g**) THC. LIPO- lipophilicity, INSOLU- insolubility, and FLEX- flexibility.

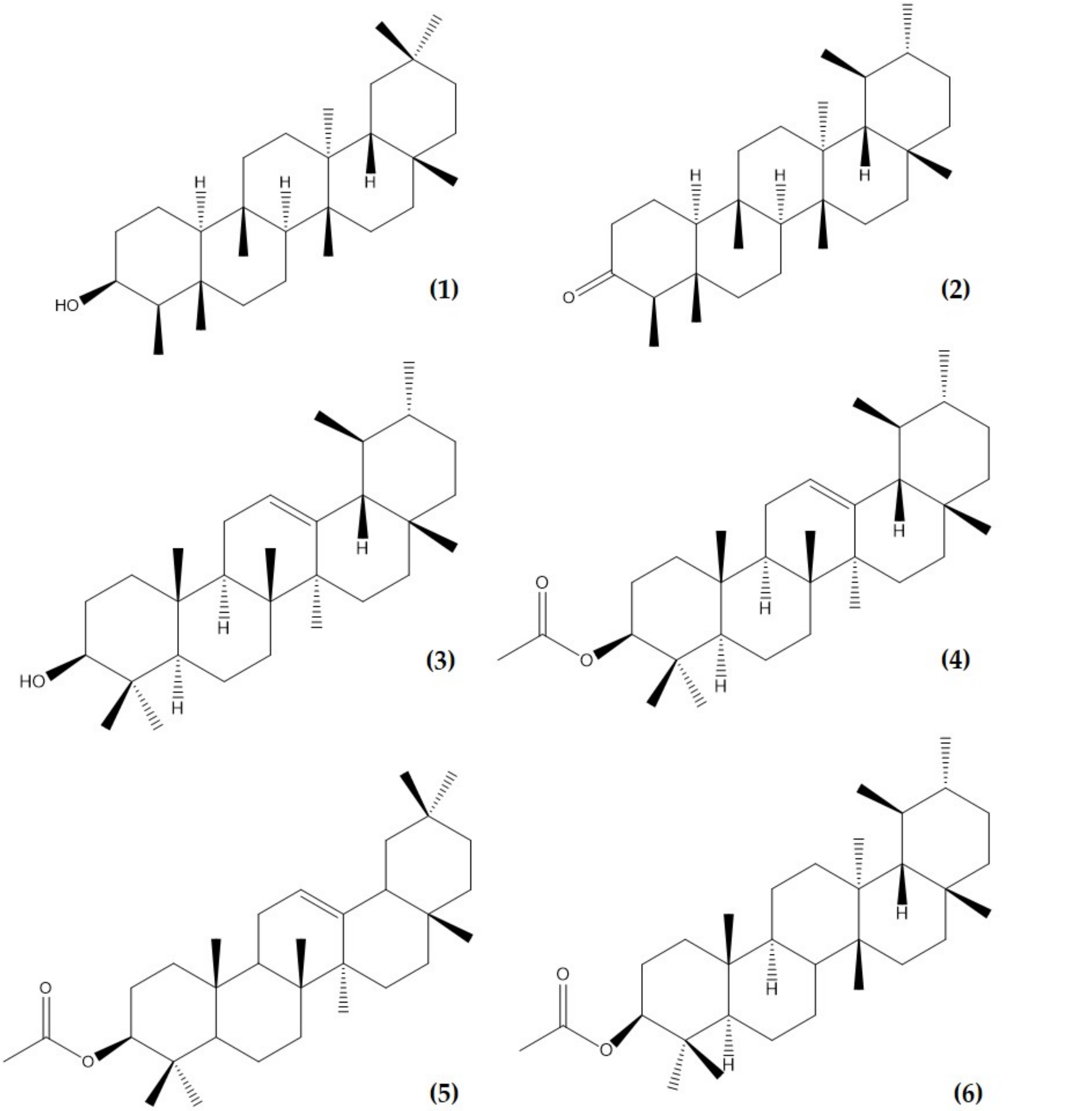

Figure 5. Structures of VP triterpenes. Epifriedelanol (**1**), friedelin (**2**), α- amyrin (**3**), α-amyrin acetate (**4**), β-amyrin acetate (**5**) and bauerenyl acetate (**6**).

Table 1. Intermolecular interactions of α-amyrin acetate, β-amyrin acetate and bauerenyl acetate with CB1.

Ligand	Binding affinity (kcal/mol)	Binding residues	Category	Type	Distance (Å)
α-amyrin acetate	–7.8	Ala118	Hydrophobic	Alkyl	3.68
		Ala118	Hydrophobic	Alkyl	3.42
		Ile119	Hydrophobic	Alkyl	4.38
		Leu122	Hydrophobic	Alkyl	4.38
		Leu122	Hydrophobic	Alkyl	5.23
		Leu126	Hydrophobic	Alkyl	4.63
		Ile119	Hydrophobic	Alkyl	4.89
		Leu122	Hydrophobic	Alkyl	4.63
		Met384	Hydrophobic	Alkyl	4.22
		Leu126	Hydrophobic	Alkyl	5.49
		Met384	Hydrophobic	Alkyl	3.92
		Ile119	Hydrophobic	Alkyl	4.65
		Leu388	Hydrophobic	Alkyl	3.67
		Leu122	Hydrophobic	Alkyl	5.12
		Ile119	Hydrophobic	Alkyl	4.53
		Phe381	Hydrophobic	Pi-Alkyl	4.10
		Phe381	Hydrophobic	Pi-Alkyl	3.94
β-amyrin acetate	–7.3	Gln115	Hydrogen Bond	Conventional	3.06
		Leu122	Hydrophobic	Alkyl	5.23
		Met384	Hydrophobic	Alkyl	4.81
		Leu385	Hydrophobic	Alkyl	4.41
		Leu388	Hydrophobic	Alkyl	4.59
		Leu126	Hydrophobic	Alkyl	5.06
		Met384	Hydrophobic	Alkyl	4.37
		Leu122	Hydrophobic	Alkyl	5.00
		Ile119	Hydrophobic	Alkyl	4.93
		Leu122	Hydrophobic	Alkyl	4.12
		Phe381	Hydrophobic	Pi-Alkyl	5.26
		Phe381	Hydrophobic	Pi-Alkyl	4.96
		Phe381	Hydrophobic	Pi-Alkyl	5.23
		Phe381	Hydrophobic	Pi-Alkyl	4.53
Bau-erenyl acetate	–7.5	Ala120	Hydrophobic	Alkyl	3.58
		Val179	Hydrophobic	Alkyl	4.99
		Ala120	Hydrophobic	Alkyl	4.75
		Ala120	Hydrophobic	Alkyl	4.18
		Val179	Hydrophobic	Alkyl	5.37
		Phe177	Hydrophobic	Pi-Alkyl	4.57
		His178	Hydrophobic	Pi-Alkyl	4.56
		His178	Hydrophobic	Pi-Alkyl	4.60
		His178	Hydrophobic	Pi-Alkyl	4.95
		His178	Hydrophobic	Pi-Alkyl	4.52
